# The *foxa2* Gene Controls the Birth and Spontaneous Degeneration of Dopamine Neurons in Old Age 

**DOI:** 10.1371/journal.pbio.0050325

**Published:** 2007-12-11

**Authors:** Raja Kittappa, Wendy W Chang, Rajeshwar B Awatramani, Ronald D. G McKay

**Affiliations:** 1 Laboratory of Molecular Biology, National Institute of Neurological Disorders and Stroke, National Institutes of Health, Bethesda, Maryland, United States of America; 2 Department of Neurology, Northwestern University School of Medicine, Chicago, Illinois, United States of America; Baylor College of Medicine, United States of America

## Abstract

Parkinson disease affects more than 1% of the population over 60 y old. The dominant models for Parkinson disease are based on the use of chemical toxins to kill dopamine neurons, but do not address the risk factors that normally increase with age. Forkhead transcription factors are critical regulators of survival and longevity. The forkhead transcription factor, foxa2, is specifically expressed in adult dopamine neurons and their precursors in the medial floor plate. Gain- and loss-of-function experiments show this gene, *foxa2*, is required to generate dopamine neurons during fetal development and from embryonic stem cells. Mice carrying only one copy of the *foxa2* gene show abnormalities in motor behavior in old age and an associated progressive loss of dopamine neurons. Manipulating forkhead function may regulate both the birth of dopamine neurons and their spontaneous death, two major goals of regenerative medicine.

## Author Summary

The restoration of dopamine neurons is a major focus of stem cell biology and regenerative medicine. The gradual loss of these neurons is a hallmark of Parkinson disease. Dopamine neurons in the midbrain convey important sensory and motor functions to the forebrain. We show that the transcription factor FOXA2 plays a central role in the birth and death of dopamine neurons in the midbrain. By defining their precursors in the ventral midbrain, we show that dopamine neurons are derived from organizer cells in the floor plate (the ventral cells of the neural tube, the embryonic foundation of the central nervous system). We also show that FOXA2 specifies the floor plate and induces the birth of dopamine neurons. Mice with only a single copy of the *foxa2* gene acquire motor deficits and a late-onset degeneration of dopamine neurons. This spontaneous cell death preferentially affects neurons associated with Parkinson disease. This work provides new strategies to generate neurons in the laboratory and to block their death in old age.

## Abstract 3

The connection between development and neurodegeneration is emphasized via a new mouse knockout of a transcription factor that is critical for dopamine neuron specification, which produces a late-onset, asymmetric degenerative condition in a manner very similar to human Parkinson disease.

## Introduction

Midbrain dopamine neurons play important roles in motor control, reward, addiction, attention, and cognition [1,2]. A progressive loss of dopamine neurons is a defining feature of Parkinson disease. This disease will cause increased hardship in many countries, as 30% of the population will be over the age of 65 y around 2025 [3]. Dopamine neurons are normally generated in limited numbers, for a restricted time, in a small region of the embryonic midbrain [4]. To improve access to these neurons, techniques have been developed to derive them from precursors dissected from the fetal midbrain and from pluripotent embryonic stem (ES) cell lines [5–11]. Despite this effort, our knowledge of the mechanisms controlling the birth and death of these cells is still limited.

Foxa2 is a forkhead transcription factor known to play a critical role in the early development of the endoderm and midline structures, including the notochord and floor plate [12–16]. FOXO transcription factors are closely related to the *foxa* genes and have a central role in cell survival, cancer, and the longevity of organisms [17–19]. Here, we show that midbrain dopamine neurons are derived from the floor plate and that foxa2 plays a central role in specifying dopamine neurons. Late in life, *foxa2* heterozygous mice spontaneously develop significant motor problems and an associated late-onset degeneration of dopamine neurons. The initial deficit is asymmetric and preferentially affects dopamine neurons of the substantia nigra (SN) while leaving the ventral tegmental area (VTA) intact, a pattern of sensitivity also seen in Parkinson patients [20].

## Results

**Figure 1:**
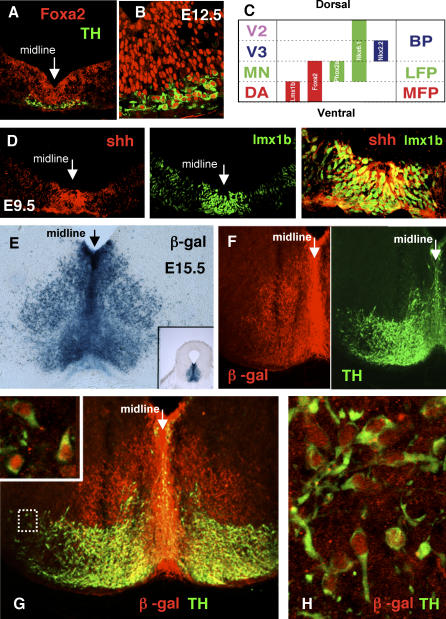
Floor Plate Origin of Dopamine Neurons (A and B) Initial differentiation of dopamine neurons is in the medial domain of the *foxa2*-expressing floor plate. Low (A) and high (B) magnification images of E12.5 midbrain stained for FOXA2 (red) and tyrosine hydroxylase (green). Note that the newly born dopamine neurons continue to express *foxa2*. (C) Schematic of the ventral progenitor domains of the embryonic mesencephalon. Dopamine precursors (DA) occupy the ventral-most position in the mesencephalon, the medial floor plate (MFP), whereas the lateral floor plate (LFP) is comprised of oculomotor neuron precursors (MN). V3 precursors are on either side of the floor plate, in the ventral basal plate (BP). V3 precursors are not the most-ventral neuronal progenitor in the midbrain, as they are in the spinal cord, because mesencephalic floor plate cells undergo neurogenesis. The expression of LMX1b, PHOX2a, and NKX2.2 expression are shown in Figures S2 and S3. NKX6.1 expression in the midbrain is shown in Figure S3. (D) SHH (red) and LMX1b (green) are coexpressed in the floor plate of the embryonic midbrain. (E) ß-galactosidase staining in the E15.5 midbrain of a *shh-cregfp:: ROSA26* mouse fetus. In this mouse, ß-galactosidase is expressed in the shh-expressing cells of the floor plate and their derivatives. Inset contains a low-power image of a coronal section of the midbrain showing that the blue ß-galactosidase signals is restricted to the most ventral region. (F–H) Immunohistochemical localization in (F) and (G) shows that both TH (green) and ß-galactosidase (red) are expressed in the same cells, demonstrating a floor plate origin for dopamine neurons. (G) Inset: enlargement of boxed cells showing colocalization of TH and ß-galactosidase at the single-cell level. (H) Another enlarged field contains numerous neurons coexpressing TH and ß-galactosidase.

Dopamine neurons can be identified by expression of tyrosine hydroxylase (TH), the rate-limiting enzyme in dopamine synthesis. In the nervous system, *foxa2* expression is restricted to the floor plate, a specialized ventral region that regulates the differentiation of nearby neurons by secreting the morphogenic signal sonic hedgehog (SHH) [14,15]. The newly generated dopamine neurons form a band at the most ventral edge of the midbrain and express FOXA2 in their nuclei (Figure 1A and 1B). A panel of antibodies against transcription factors was used to define the domains of neuronal precursors in the ventral midbrain (Figure S1). The expression of the transcription factors LMX1b, NKX2.2, and PHOX2a between embryonic day (E)9.5 and 11.5 defines three adjacent ventral domains of neural progenitors before the first dopamine neurons are formed (Figure 1C). The transcription factor LMX1b is expressed in the most ventral domain, the medial floor plate, continuously from E9.5 through E11.5 (Figure S1C–S1E).

SHH is coexpressed with LMX1b in the floor plate (Figure 1D). From previous studies, it is unclear whether SHH-expressing cells are the direct precursors to dopamine neurons or if they are derived from a more lateral precursor that is induced by signals from the floor plate [21–23]. Genetic tracing using Cre-recombinase expressed from the shh regulatory sequences (*shh-creR26R* mice) allows the derivatives of these most medial cells to be identified [24]. When *shh-cre* mice were crossed with the R26R reporter strain, the expression of ß-galactosidase is surprisingly found outside the midline (Figure 1E and inset). At E15.5, when the majority of dopamine neurons have become postmitotic, they express TH and also express ß-galactosidase, definitively demonstrating that midbrain dopamine neurons are derived from the medial floor plate (Figure 1F–1H).

**Figure 2:**
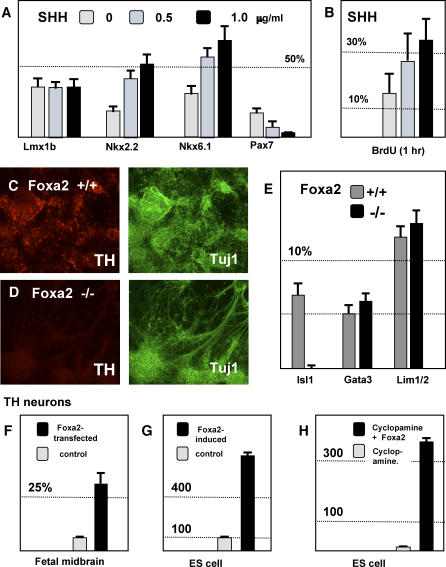
The In Vitro Role of *foxa2* and SHH in Cell Fate Specification in the Ventral Mesencephalon (A) Effects of Shh on E8.5 mesencephalic precursors in vitro. In the absence of exogenous SHH, many LMX1b^+^ precursors (36.59 ± 6.10%) were observed. NKX2.2^+^ (19.83 ± 4.39%), NKX6.1^+^ (30.47 ± 6.72%), and PAX7^+^ precursors (16.33 ± 5.02%) were present. In the presence of 500 ng/ml of SHH protein, the proportion of NKX2.2^+^ (41.84 ± 5.84%) and NKX6.1^+^ (56.62 ± 7.04%) cells increased, whereas, the proportion of PAX7^+^ cells diminished (6.89 ± 2.54%). Despite these effects on other cell types, the proportion of LMX1b^+^ dopamine precursors was stable (32.48 ± 5.56%). Higher concentrations of SHH would be expected to further ventralize the midbrain precursors at the expense of more dorsal fates. In the presence of 1 μg/ml of SHH protein, there was a further increase in NKX2.2^+^ and NKX6.1^+^ cells (nkx2.2^+^, 52.75 ± 6.34%; nkx6.1^+^, 69.12 ± 8.73%) and a greater reduction of PAX7^+^ cells (2.24 ± 0.81%). At this high concentration of SHH, the percentage of dopamine precursors again remained unchanged (33.04 ± 6.61%). (B) SHH has a proliferative effect on mesencephalic precursors in cell culture. BrdU was added to day 3 cultures of mesencephalic precursors, grown in different concentrations of SHH protein. BrdU was added for 1 h before fixation and staining. The percentage of cells incorporating BrdU increased with the concentration of SHH in the culture. (C) Wild-type mesencephalic explants differentiate to generate a large number of neurons (Tuj1, green) and a significant proportion of these neurons express tyrosine hydroxylase (TH, red). (D) Mesencephalic explants from *foxa2^−/−^* embryos also generate many neurons, but TH expression is absent. (E) *foxa2^−/−^* explants similarly do not yield islet-1–expressing motor neurons although the differentiation of GATA3- and LIM1/2-positive neurons born outside of the floor plate is unaffected. The developmental expression pattern of these proteins can be seen in Figure S3. (F) Overexpression of *foxa2* in cultured E10.5 mesencephalic explants results in an increase in TH-expressing dopamine neurons. (G) Doxycycline induction of *foxa2* in differentiating F4 mouse embryonic stem cells significantly increases the number of resulting dopamine neurons. (H) Inhibition of SHH signaling by cyclopamine suppresses dopaminergic differentiation of ES cells. The induction of *foxa2* by doxycycline overcomes cyclopamine suppression of dopamine neuron differentiation.

For the efficient ex vivo generation of dopamine neurons, it is important to understand the mechanisms that induce dopamine neuron identity. SHH gives increased numbers of dopamine neurons in midbrain primary explants and in neuronal cultures derived from ES cells [8,22,23]. This effect is thought to reflect the morphogenic induction of the dopaminergic fate. Access to reagents that define distinct precursor types allows the morphogenic effects of SHH in vitro to be reexamined. When dissociated cells from the developing ventral midbrain (E8.5) were exposed to increasing concentrations of SHH, the proportion of LMX1b^+^ cells did not change, but the proportion of the NKX2.2^+^ and NKX6.1^+^ cells increased while the more dorsal PAX7 expression decreased (Figures 2A and S2). This result shows that, under these conditions, SHH does not induce the precursors of dopamine neurons, whereas the proportion of more lateral precursors is altered in the graded way expected of a morphogenic signal. However, SHH is also a mitogen for neural precursors and this mitogenic effect may account for the increased number of dopamine progenitors (Figure 2B). These results suggest that growth control systems for dopamine neuron precursors are distinct from most other ventral midbrain neuron types.

A consequence of these results is that production of dopamine neurons in the laboratory will be limited by the number of floor plate cells. As *foxa2* is known to control the generation of the floor plate, we asked whether this gene controls the number of TH-positive cells in vitro. Because *foxa2*-null embryos do not survive past E10.5, E8.5 midbrain explants from *foxa2* homozygous mutant (*n* = 8) and control embryos (*n* = 12) were placed in culture. Neural progenitors migrate out from the explant and differentiate into Tuj1-positive neurons. In all of the wild-type explants, TH^+^ neurons were abundant (Figure 2C). In contrast, the mutant explants generated no TH^+^ neurons (Figure 2D). Using transcription factor expression, the neuron types in the ventral midbrain were distinguished (Figure S3). The islet1-positive oculomotor neurons were also absent, but more-lateral neurons expressing GATA3 and LIM1/2 were generated in cultures from *foxa2^−/−^* embryos (Figure 2E). These data show that the *foxa2* gene is specifically required for the generation of dopamine and motor neurons, the two neuron types derived from the floor plate.

To ask whether expression of *foxa2* would cause an increase in the number of dopamine neurons generated in vitro, a *foxa2* expression plasmid was transfected into E10.5 midbrain cells. A 4-fold increase in the proportion of TH^+^ cells was observed (Figure 2F). A mouse embryonic stem (mES) cell line engineered to inducibly express a *foxa2* transgene generated 7-fold more TH^+^ cells (Figure 2G). The SHH antagonist, cyclopamine, inhibited the production of TH^+^ neurons in vitro. This effect was reversed upon induction of *foxa2* even in the presence of cyclopamine (Figure 2H). These results are consistent with an early role for shh in dopamine neuron specification demonstrated in mice carrying a conditional mutation in the SHH receptor *smoothened* [25]. Genetic analysis in zebrafish also demonstrates a role for *foxa2* in the specification or patterning of ventral neurons in the midbrain and hindbrain [26]. Our data suggest that *foxa2* specifies dopamine neurons in mammals.

**Figure 3:**
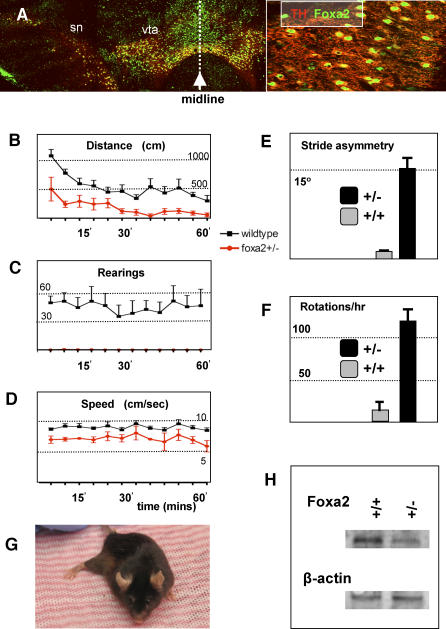
*foxa2* and the Adult Midbrain (A) Coronal section through adult mouse midbrain stained showing that *foxa2* is expressed in dopamine neurons of the SN and VTA of the adult mouse midbrain. ([A], inset) Enlargement of cells in (A) showing colocalization of TH and FOXA2 at the single cell level. (B–E) Analyses of motor behavior in mutant and wild-type mice. (B) Horizontal movement of mutant (red) and wild-type (blue) mice in the activity monitor. (C) Vertical movement (rearing) of mutant (red) and wild-type (blue) mice in the activity monitor. Note that mutant mice do not rear at all. (D) Speed of mutant (red) mice is compromised relative to wild-type (blue) mice. (E) Footprint analysis was used to quantitate the angle of spinal curvature in mutant mice and age-matched wild-type mice. (F) Amphetamine significantly induces rotational movement in *foxa2^+/−^* as compared to wild-type controls. (G) Image of 18-mo-old *foxa2^+/−^* mouse with a rigid right rear limb. (H) Western analysis of matched 1-mm sections of ventral midbrain shows a reduction of FOXA2 protein in *foxa2^+/−^* mice as compared to wild-type controls. As a loading control for total protein, beta-actin staining is shown below.

Dopamine neurons in the adult brain continue to express *foxa2* (Figure 3A). A *foxa2* null allele was crossed into the C57BL/6 strain because this background is widely used in physiological and behavioral studies. Mice entirely lacking *foxa2* die in early development. In all the data reported here, the effect of a single copy of the *foxa2* gene, haploinsufficiency, was assessed in a C57BL/6 background. Haploinsufficient mice spontaneously developed major motor abnormalities. These behaviors were first observed at 18 mo of age when mice present with an asymmetric posture associated with a muscular rigidity that progresses from the tail, through the hind limbs, to the trunk. *foxa2^+/−^* animals showed a slower speed of movement but reduced horizontal movement and a complete loss of vertical movement (Figure 3B–3D, *foxa2^+/+^ n* = 6, *foxa2^+/−^ n* = 4; Video S1). The mice demonstrate a unilateral constriction/torsion of the trunk so the spine becomes curved towards either the left or right side. In a group of affected animals, the spinal curvature was 15° measured by analysis of footprints (Figure 3E). Occasional episodes of high-frequency tremoring have been observed. Rigidity and loss of mobility in the hindlimbs become so severe that the limb is abnormally extended or splayed (Figure 3G).

These defects could be caused by deficits in non-dopaminergic systems. A widely used and quantitative behavioral assay of dopaminergic function in rodents is amphetamine-induced rotation [27]. Rats or mice lesioned acutely and unilaterally with 6-OHDA circumambulate ipsilaterally in response to amphetamine, and the extent of rotational movement is directly correlated with the severity of the lesion. We assayed amphetamine-induced rotational behavior in old *foxa2^+/−^* and wild-type C57BL/6 mice. A significant increase in rotational movement was observed in *foxa2* mutants (Figure 3F). Rotations occurred in the clockwise or counterclockwise direction, depending on the individual mouse, and were ipsilateral to the “kinked” side of the mouse. In the ventral midbrain of 1-y-old heterozygous animals, there is a partial loss of FOXA2 protein, suggesting that a reduced level of the FOXA2 protein leads to a behavioral deficit that spontaneously appears in old age (Figure 3H).

**Figure 4:**
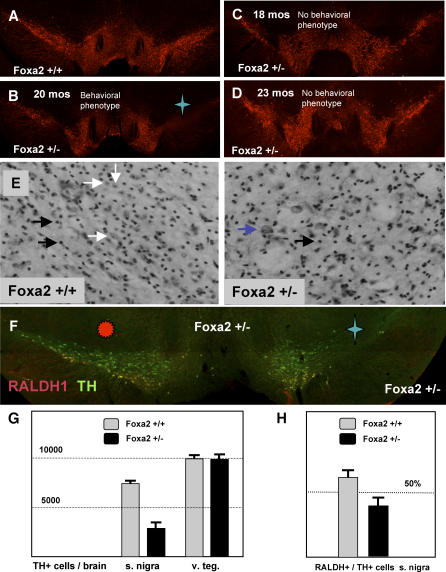
Dopamine Neuron Loss in *foxa2^+/−^* Mice (A and B) Dopamine neurons in a 24-mo-old wild-type mouse (A) and a *foxa2^+/−^* littermate (B). The mouse in (B) possessed a kinked posture and the other late-onset motor phenotypes. A significant loss of nigral dopamine neurons is easily observed on one side of the brain marked by a blue star in the *foxa2^+/−^* mouse. (C and D) TH staining of the the ventral midbrain in older *foxa2^+/−^* animals without late onset behavioral abnormalities. Note that the dopaminergic system resembles the wild-type condition seen in (A). (E) Nissl staining of wild-type and *foxa2^+/−^* mutant mice. Representative glia and neurons are highlighted with white and black arrows, respectively. Note the significant loss of normal neuronal morphologies in the mutant animal. Occasionally, a dysmorphic cell, highlighted here with a purple arrow, can be seen in the mutant, but not the wild-type brain. (F) Ventral tier dopamine neurons, stained for RALDH1 (red), are selectively lost in *foxa2^+/−^* animals. In this animal, nigral dopamine neurons are almost completely lost on the left side (marked by the blue star), whereas the right side (marked by the red sun) is much less affected (note that these are coronal sections, presented as if the animal were facing the viewer, so that the left side of the animal is on the viewer's right side. (G) Quantitation of dopamine neurons in the SN and VTA of wild-type (*n* = 3) and *foxa2^+/−^* (*n* = 3) mice. The number of dopamine neurons in the mutant SN (2,765.0 ± 750.5) is substantially smaller than in the control SN (7,344.3 ± 197.9). In contrast, the number of the dopamine neurons in the VTA is roughly the same in wild-type (9,922.0 ± 375.7) and mutant mice (10,064.7 ± 355.4). (H) The percentage of RALDH1-expressing neurons in the SN is reduced in *foxa2^+/−^* mutants as compared to wild-type controls.

The amphetamine-induced rotational behavior suggests an asymmetric loss of dopamine neurons. Immunohistochemistry for TH-positive cells was performed to shed light on potential cellular causes of the motor problems. Affected mice demonstrated an asymmetric loss of TH expression in midbrain dopamine neurons in the SN sparing the VTA (Figure 4A and 4B). No loss of dopamine neurons was seen in old *foxa2* mutants without behavioral abnormalities (Figure 4C and 4D). In the SN of affected *foxa2^+/−^* animals, there are few neurons recognized by Nissl staining (Figure 4E). Serial sections through the midbrain dopaminergic system of a single animal show an almost total loss of TH^+^ neurons in the SN on one side of the brain and much less damage to the contralateral SN (Figure S4). In *foxa2^+/−^* animals (*n* = 3) showing abnormal motor behaviors, compared to *foxa2^+/+^* age-matched controls, there is a specific loss of SN neurons (Figure 4G). This loss of dopamine neurons occurs in one third of animals over 18 mo old, and the asymmetric cellular loss explains the amphetamine induced rotation.

Within the SN, there are different types of dopamine neurons. The most ventral neurons specifically express retinaldehyde dehydrogenase-1 (RALDH1; [28]). In *foxa2* mutant mice, there is a loss of RALDH1-positive neurons even when substantial numbers of dopamine neurons are still present in the SN. In the animal shown in Figure 4F, the SN on one side of the brain has a severe loss of dopamine neurons. In the contralateral SN, TH-positive cells are present, but there is a selective loss of RALDH1-positive cells (Figure 4F and 4H). Many of the affected animals show an asymmetric loss of dopamine neurons, and the most affected side could be either the right or left (marked by a star in Figures 4B and 4F). These data suggest the degenerative process is progressive, the RALDH1-positive neurons are the most vulnerable cells, and the SN on one side of the brain is affected first and then the disease progresses to the contralateral midbrain.

Lewy bodies, cellular inclusions immunoreactive for alpha-synuclein, ubiquitin, and other proteins, are often found in the basal ganglia and cortex of the human parkinsonian brain [29]. Pathological staining for alpha-synuclein was not observed in *foxa2* mutant mice, but a small number of TH-expressing dopamine neurons that were highly immunoreactive for ubiquitin were seen in the SN of mutant but not in wild-type C57BL/6 mice. Gliosis is an activation of astrocytes that often occurs in neurodegenerative disease [30]. Accompanying the loss of dopamine neurons in *foxa2* mutants, there is an increase in the number of activated glia measured by expression of the glial fibrillary acidic protein (GFAP) in the substantia nigra pars compacta, in the substantia nigra pars reticulata, and other regions of the ventral midbrain (Figure S5A–S5D). In contrast to neuron loss, this activation occurs in a symmetric manner.

## Discussion

Rigidity, tremor, reduced and slow movement with asymmetric behavioral features, and dopamine neuron loss are characteristically found in Parkinson patients [31]. The age-dependent motor defects and the late loss of dopamine neurons seen in *foxa2^+/−^* mice are similar to symptoms of Parkinson disease. In mammalian cells, survival is controlled by phosphorylation of FOXO proteins that respond to oxidative stress by regulating apoptosis [32.^33^]. Overexpression of *pha-4*, the nematode *foxa* homolog, in Caenorhabditis elegans induces the superoxide dismutase, sod-1, and increases longevity [34]. These data suggest that FOXA2 regulates the response of dopamine neurons to oxidative stress, but it is clear that a cell autonomous function in dopamine neurons should be explicitly assessed.

In this report, we have shown that mice heterozygous for a mutation in *foxa2* develop a late-onset condition associated with a significant loss of midbrain dopamine neurons. It will be important in the future to determine how functional interactions with other brain regions are altered as the dopamine neurons degenerate. Here, we show the neurons of the SN are more sensitive than the neurons of the VTA to the pathology in *foxa2^+/−^* mice. Asymmetric motor deficits and preferential loss of nigral neurons are characteristic in disorders of the nigrostriatal system [20]. The asymmetric degeneration of nigral dopamine neurons shows that *foxa2^+/−^* mice model features of human degenerative disease not previously seen in animal models.

Oxidative stress is considered to be a primary cause of Parkinson disease [35,36]. Recent studies have identified mutations in a number of genes (*PARK* genes) associated with Parkinson disease. These genes include *α-synuclein* [37], *parkin* [38], *DJ-1* [39], *PINK1* [40], and most recently, *LRRK2* [41,42]. Mice have been generated with mutations in many of the *PARK* genes but, surprisingly, spontaneous degeneration of dopamine neurons and motor function deficits have not been observed in these animals. The forkhead transcription factors are targets of the PI3K/Akt signaling pathway that is an important regulator of cell growth and survival [43]. Growth factors proposed as treatments for Parkinson disease also act through this pathway [44]. Recently, genes associated with human parkinsonism have been shown to be regulators of the PI3K/Akt pathway [45.^46^]. The clear cell loss reported here suggests that FOXA2 is the transcriptional effector of dopamine neuron survival. It will be interesting to analyze potential interactions between FOXA2, mutant *PARK* genes, and neurotrophic factors.

This spontaneous model is important because it will allow us to understand the dynamics of dopamine neuron degeneration. In the SN of Parkinson patients, the RALDH1-expressing ventral dopamine neurons are particularly vulnerable to the etiology of Parkinson disease [47]. An initial asymmetric motor deficit, an asymmetric dopamine loss measured by positron emission tomography (PET) scanning, and dopamine neuron pathology are characteristic features of Parkinson disease [31,48]. Most studies on whole animals that address Parkinson disease use specific toxins to kill dopamine neurons [49]. Toxins are valuable, and they can specifically target the ventral tier neurons at risk in Parkinson disease, but they inadequately address the progressive nature of the disease and the increased sensitivity with age [50]. *foxa2^+/−^* animals give us access to the normal mechanisms that initiate and sustain loss of neurons in the SN. There would be great clinical benefit if the progression of disease could be slowed when patients are first diagnosed.

We present data that address the related questions: how to make dopamine neurons efficiently in the laboratory and how to support their survival in the brain. We present a precise map of the distinct origin of dopamine neurons that is fundamental to solving a major current problem in cell therapies for Parkinson disease, the identity of dopamine neurons in the graft. However, our data have a significance that goes beyond the technology of cell therapy. Stem cell biology is often criticized for the slow transition to explicit clinical benefit. The work presented here illustrates how a developmental approach that is implicit in stem cell biology leads to a focus on the mechanisms that control the origin and survival of the cell of interest. In the case of dopamine neurons, our data suggest a central role for the PI3K/Akt/Fox survival pathway in defining the origin of dopamine neurons and developing an effective response to Parkinson disease.

## Materials and Methods

### Immunohistochemistry and histology.

Timed pregnant CD-1 mice (Charles River Laboratories) were obtained and embryos were manually dissected and fixed in 4% paraformaldehyde (PFA) in PBS, transferred to 20% sucrose overnight, and embedded in O.C.T compound (Tissue Tek, Sakra). 12-μm sections were cut on a Microm MH 500 OM cryostat and processed for immunohistochemistry. Sections stained with the anti-SHH antibody were treated with 50 mM NH4Cl for 20 min at room temperature and washed twice with PBS prior to incubation with the primary antibody.

Adult mice were anesthetized with pentobarbital sodium (Nembutal) and perfused with 20 ml of PBS followed by 40 ml of 4% PFA. Brains were dissected out, transferred to 20% sucrose overnight, and 40-μm sections were cut on a cryostat and maintained as floating sections in PBS. Immunostaining was visualized on a confocal microscope. All animal work was carried out in accordance with National Institute of Neurological Disorders and Stroke (NINDS) Animal Studies Protocols 1204–05, 1205–05, and 1247–05, as approved by the NINDS Animal Care and Use Committee. Cell counts were performed on every sixth section within the midbrain, directly at the microscope. In cases in which cell density made it difficult to easily discern individual cells, cell bodies stained for TH, RALDH1, and GFAP were matched to their DAPI-labeled nuclei. To estimate the absolute number of midbrain dopamine neurons, we multiplied the total number of cells by six (sampling frequency) and made the Abercrombie correction. We measured the nuclei of 50 midbrain dopamine neurons, both in the SN and VTA, and determined the mean nuclear diameter to be 8.69 μm, which we used for the Abercrombie correction.

For Nissl staining, 40-μm brain sections were washed in distilled water and mounted on slides. Sections were treated with cresyl violet for 5 min and then washed again in distilled water. Slides were then run through an ethanol series (50%, 70%, 90%, 95%, and 100%) and finally dipped in xylene for 2 min, before mounting in Permount.

### Antibodies.

For immunohistochemistry of mouse embryo sections and fixed cells in vitro, the following antibodies were used: β-galactosidase (1:500, rabbit monoclonal; Biogenesis); BrdU (1:200, rat monoclonal; Axyll); FOXA2, Islet1, LIM1/2, NKX2.2, PAX7, and SHH (1:10, mouse monoclonal; Developmental Studies Hybridoma Bank, University of Iowa); FOXA2 (1:4,000, rabbit polyclonal; gift of Ariel Ruiz I Altaba); FOXA2 (1:50, goat polyclonal; Santa Cruz Biotechnology); GATA3 (1:200, mouse monoclonal; Santa Cruz Biotechnology); GFAP (1:500, rabbit polyclonal; DAKO); LMX1b (1:4,000, guinea pig polyclonal; gift of T. Jessell); LMX1b (1:500, rabbit polyclonal; gift of T. Muller); nestin (1:500, rabbit polyclonal; McKay lab); NKX2.2 (1:400, guinea pig polyclonal; gift of B. Sosa Pineda); NKX6.1 (1:1,000, rabbit polyclonal; gift of M. German); PTX3 (1:400, rabbit polyclonal; Upstate); RALDH1 (1:100, rabbit polyclonal; gift of Greg Duester); Phox2a (1:800, rabbit polyclonal; gift of J.-F. Brunet); SOX1 and SOX2 (1:500, rabbit polyclonal. gift of R. Lovell-Badge); and tyrosine hydroxylase (1:500, rabbit and sheep polyclonals; Pel-freez).

### Cell culture.

E8.5 embryos (9–13 somites) were obtained from timed pregnant CD-1 mice (Charles River Laboratories). Embryos were incubated in N2 medium containing 1% amylase (Sigma) for 1 h at 37 °C, to facilitate manual dissection. After removal of ectoderm and head mesenchyme, embryonic midbrains were moved to amylase-free N2 medium. Pooled tissue was washed 3× in PBS and then digested in N2 medium containing 0.005% Trypsin-EDTA (Gibco) for 5 min at room temperature. Dissociated mesencephalic cells were added to N2 medium containing Trypsin Inhibitor (Sigma), 100 ng/ml FGF2, 100 ng/ml (FGF8), and 500 ng/ml Shh (R&D Systems). Three embryo equivalents of mesencephalic cells were plated per well in a 24-well plate (Costar), coated with poly-L-ornithine (Sigma) and fibronectin (R&D Systems). Upon cell attachment, the medium was changed to N2 medium (without trypsin inhibitor) containing 100 ng/ml FGF8 and shh. Cells were fixed with 4% PFA after 4 d of expansion. To assay cell proliferation, cells were treated with 10 μM BrdU (Roche) for 1 h prior to fixation. Four independent experiments were performed in which each condition was analyzed in duplicate wells.

CNS explants were dissected from E8.5 *foxa2^−/−^* embryos and wild-type embryos. Explants were plated directly in N2 medium containing 100 ng/ml FGF8 and 500 ng/ml shh, as described above. After 4 d in culture with mitogens, the explants were grown for four additional days in the absence of exogenous growth factors to promote differentiation. After 4 d of differentiation, the explants were fixed with 4% PFA.

For overexpression of *foxa2*, E10.5 embryonic midbrain cells were harvested and plated in a manner identical to the E8.5 experiments, above, with the exception that two embryo equivalents of mesencephalic cells were plated per well of a 24-well plate (Costar). Cells were transfected, using the lipofectamine reagent (Invitrogen), with a CMV-foxa2 vector (generous gift of R.J. Matusik) and with a GFP expression vector (gift of T. Misteli, National Cancer Institute).

The mouse embryonic stem cell line, F4, which expresses *foxa2* inducibly, under the control of doxycycline, will be described in greater detail elsewhere (A. Kuzmichev and R. D. G. McKay, unpublished data). Briefly, these cells were generated by homologous recombination into the HPRT locus using a vector modified from a previously described vector [51] in which the *oct4* transgene was replaced by *foxa2*. We differentiated the mES cells to neurons using a method modified from a previously described protocol [52]. Approximately 50,000 F4 cells were plated per well of a fibronectin-coated, 24-well plate into N2 medium. The cells were differentiated for 28 d in N2, in the absence of exogenous growth factors. *foxa2* was induced using doxycycline from days 6–23 of culture (no induction for the first and last 5 d of the differentiation).

### Behavior.

Spontaneous motor activity was analyzed using the Versamax Animal Activity Monitoring System (AccuScan Instruments). Animals were placed into the 16.5” by 16.5” chamber for 60 min where vertical and horizontal movements detected by infrared beams were analyzed by VersaMax software. When wild-type and mutant mice were compared, there was no simple difference in the time spent in the center of the chamber. For amphetamine-induced rotation, mice were injected with 2.5 mg/kg of amphetamine intraperitoneally, and clockwise and counterclockwise rotations were measured using the Rota-count 8 system (Columbus Instruments) for 60 min.

## Supporting Information

Figure S1 Expression of Transcription Factors in Ventral Midbrain Progenitors (A–H) Transverse sections through mouse mesencephalon at E9.5 (C) and (F), E10.5 (A), (B), (D), and (G), and E11.5 (E) and (H), stained with anti-LMX1b (green in [A], [C], and [E]; red in [D]), anti-PHOX2a (red in [A]; green in [B] and [F–H]), anti-NKX2.2 (red in [B]), and anti-FOXA2 (red in [C], [E], and [F–H]; green in [D]) antibodies. Double-staining is indicated by yellow.
(A) LMX1b expression (dopamine precursors) occurs in a single domain at the ventral midline. PHOX2a^+^ cells (oculomotor neuron precursors) are observed in an immediately adjacent, nonoverlapping domain.
(B) NKX2.2-expressing cells (V3 neural precursors) occupy the domain immediately dorsal to the motor neuron precursor domain. Some expression of NKX2.2 can be observed in PHOX2a-expressing domain.
(C–E) Dopamine precursors express the floor plate marker, FOXA2, and are found in the medial part of this domain. LMX1b^+^ cells are found in the medial FOXA2^+^ domain.
(F–H) PHOX2a^+^ cells are found in the lateral parts of the FOXA2^+^ domain. Numerous PHOX2a^+^ precursors coexpress FOXA2 at E9.5 (F). As these cells migrate to the pial surface, they down-regulate FOXA2 (G) and (H).
(1.1 MB PDF)

Figure S2 Expression of *nkx* Genes in Relation to the Mesencephalic Floor Plate Transverse sections through mouse mesencephalon at E9.5 (B), E10.5 (A) and (C), and E11.5 (D), stained with anti-FOXA2 (green in [A]; red in [B–D]), anti-NKX2.2 (red in [A]), anti-NKX6.1 (red in [B–D]) antibodies. The NKX2.2-expressing cells in (A) flank the FOXA2^+^ floor plate in the midbrain, just as they do in the developing spinal cord.
(695 KB PDF)

Figure S3 Expression of Transcription Factors in Neurons of the Ventrolateral Fetal Midbrain (A) GATA3 expression is observed in a subset of neurons differentiating in the NKX6.1 domain E10.5.
(B and C) At E11.5, GATA3^+^ neurons are located immediately adjacent to the NKX6.1^+^ motoneurons (B). Like GATA3, LIM1/2 is expressed in neurons immediately adjacent to the NKX6.1-expressing motor neurons (C). In (B) and (C), NKX6.1 expression is lost as progenitors differentiate to GATA3- and LIM1/2-positive neurons.
(D) At E12.5, motor neurons are identified by PHOX2a expression, and more lateral neurons by LIM1/2 expression. LIM1/2 expression at E12.5 expands dramatically, compared to E11.5 (C).
(461 KB PDF)

Figure S4 Gliosis in *foxa2^+/−^* Mice (A) Widespread gliosis is seen in the ventral midbrain of *foxa2^+/−^* mice (TH, green; GFAP, red).
(B and C) Increased density of glia is easily seen in the red nucleus of wild-type (B) and *foxa2^+/−^* mice (C).
(D) Quantitation of glia in wild-type and *foxa2^+/−^* dopaminergic system throughout the VTA, the SN pars compacta and reticulata, and red nucleus.
(2.3 MB PDF)

Figure S5 Severe Asymmetric Loss of Nigral Dopamine Neurons in One Mouse Coronal sections through the midbrain of a wild-type (A), (C), (E), (G), and (I) and a *foxa2^+/−^* (B), (D), (F), (H), and (J) mouse at the indicated level. There is an almost complete loss of nigral dopamine neurons on the left side, whereas the right side is relatively intact, quantitated in (K).
(684 KB PDF)

Video S1 *foxa2* Mutant with Late-Onset Motor Phenotype This is a 20-mo-old *foxa2* heterozygote in a C57BL/6 background that has developed the late-onset motor problems characterized, here. Note the asymmetric kinked posture and the labored gait in this mouse.
(1.3 MB MPG)

## Abbreviations

E: embryonic day
ES: embryonic stem
GFAP: glial fibrillary acidic protein
shh: sonic hedgehog
SN: substantia nigra
TH: tyrosine hydroxylase
VTA: ventral tegmental area
