# Supplementary material for: The foxa2 Gene Controls the Birth and Spontaneous Degeneration of Dopamine Neurons in Old Age
Source: PLoS Biol. 2007 Dec 11;5(12):e325. doi: 10.1371/journal.pbio.0050325 (PMC2121110; doi:10.1371/journal.pbio.0050325)
Supplement: Supplementary file 1 [file pbio.0050325.sg001.pdf]

Supplementary Figure 1   Kittappa et al.

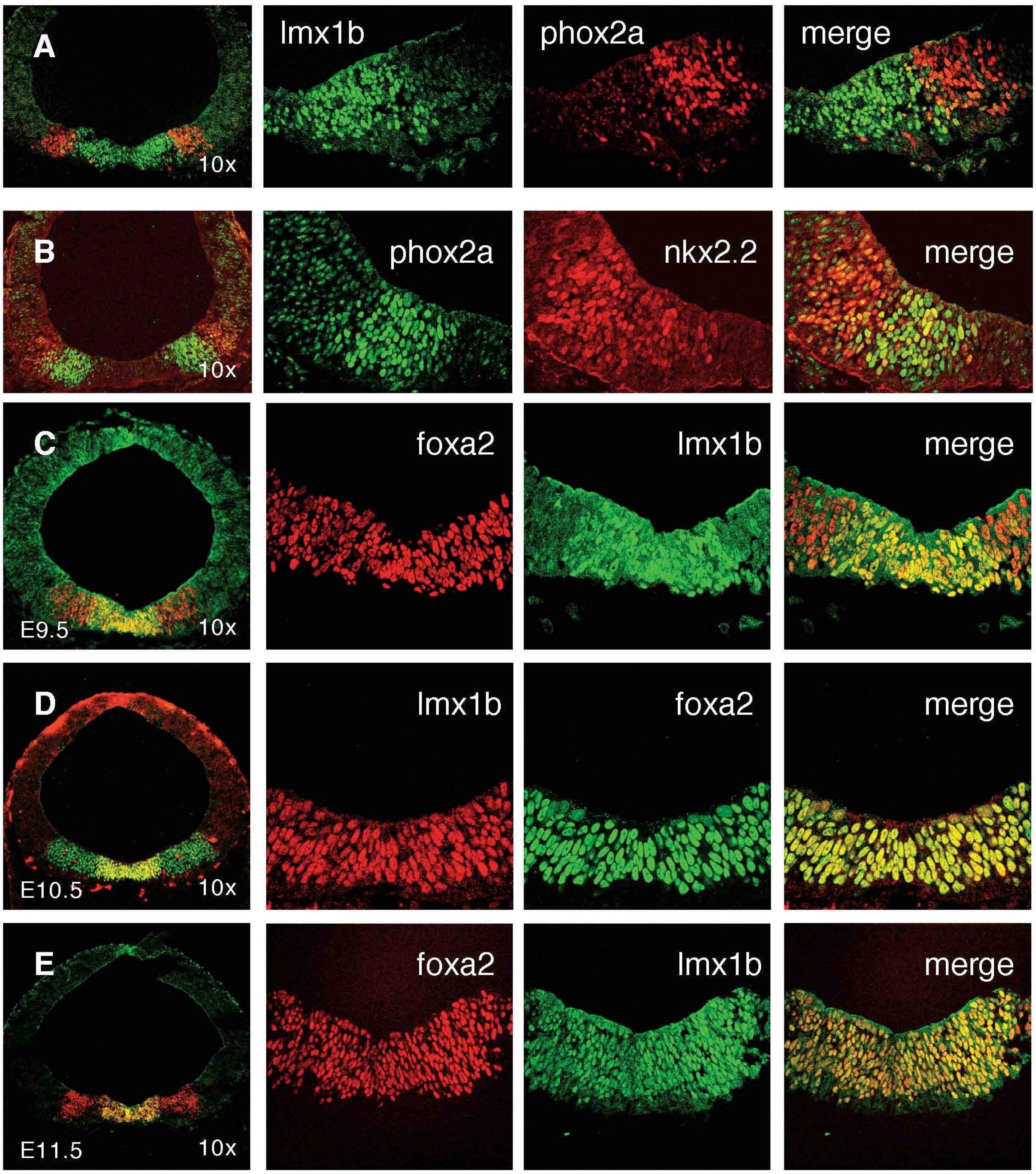

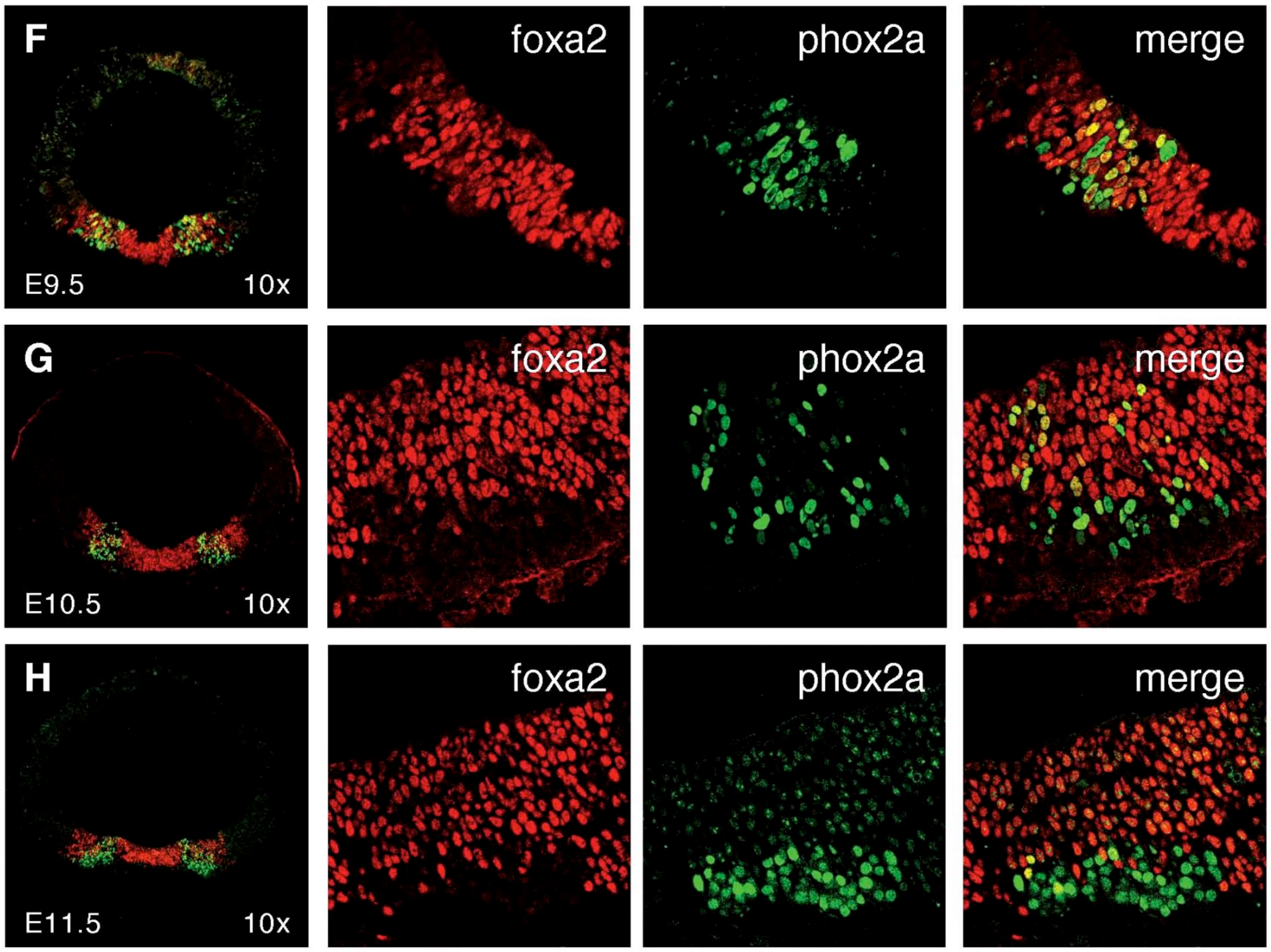
F
E9.5
10x
foxa2
phox2a
merge
G
E10.5
10x
foxa2
phox2a
merge
H
E11.5
10x
foxa2
phox2a
merge
