# Supplementary material for: The foxa2 Gene Controls the Birth and Spontaneous Degeneration of Dopamine Neurons in Old Age
Source: PLoS Biol. 2007 Dec 11;5(12):e325. doi: 10.1371/journal.pbio.0050325 (PMC2121110; doi:10.1371/journal.pbio.0050325)

Supplementary Figure 2 Kittappa et al.

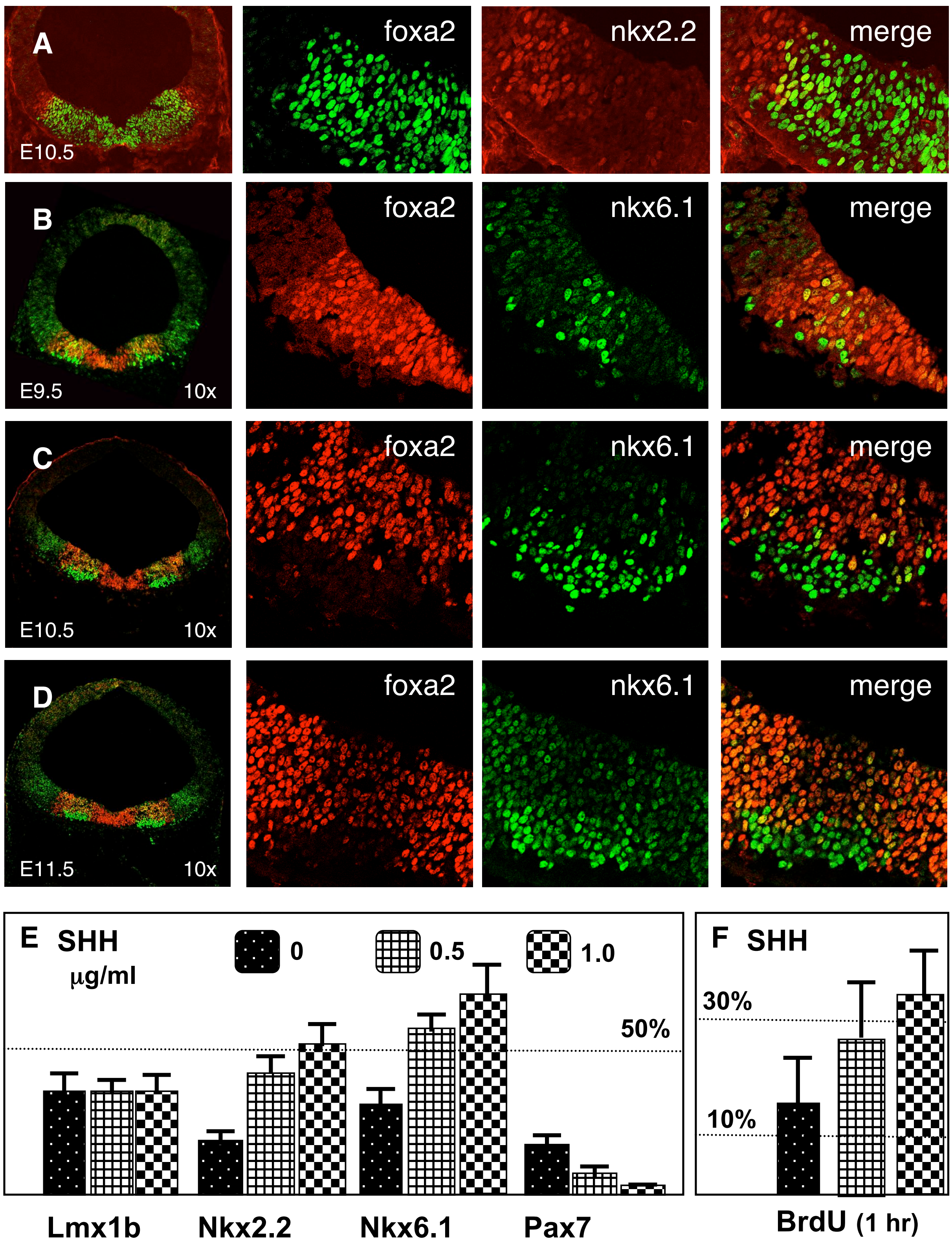

Supplement: Supplementary file 2 [file pbio.0050325.sg002.pdf]
